# Attitude of athletes towards doping: A dilemma in Pakistan

**DOI:** 10.12669/pjms.36.7.1922

**Published:** 2020

**Authors:** Ghulam Shabbir Anjum, Nazia Mumtaz, Ghulam Saqulain

**Affiliations:** 1 Ghulam Shabbir Anjum, M Phil (Sports Sciences & Health Physical Education), National Athletics Coach, Pakistan Sports Board, Islamabad, Pakistan; 2 Dr. Nazia Mumtaz, PhD (Rehabilitation Sciences), Head of Department, Department of Speech Language pathology, Faculty of Rehab and Allied Health Sciences, Riphah International University, Lahore, Pakistan; 3 Dr. Ghulam Saqulain, F.C.P.S (Otorhinolaryngology) Head, Department of Otorhinolaryngology, Capital Hospital PGMI, Islamabad, Pakistan

**Keywords:** Attitudes, Anti-doping, Performance enhancing drugs, Sports

## Abstract

**Objectives:**

To evaluate the attitude and of athletes towards performance enhancement through doping and leading reason of their decision for the use of doping in a country.

**Methods:**

This Cross-Sectional descriptive study was conducted with non-probability convenience sampling over a period of six months from November 2018 to May 2019. This study included n=377 National and international athletes/players, of both genders aged 17-35 years, camping for preparation of 13^th^ South Asian Games 2019 at Pakistan Sports Board, Jinnah Complex Islamabad, Pakistan. The athletes/ players with any disease, trauma or working as coaches or officials were excluded. Basic demographic sheet and Athletes Attitude to Doping Questionnaire were used for data collection which was analyzed using SPSS 21.

**Results:**

Study revealed significant difference in the Mean and Median scores of the six anti-doping factors with very low scores for “Long Term Health Implications” (Mean= 2.14, Md=2) and “Psychosocial Influences” (Mean=3, Md=3) compared to a high score for the remaining factors, indicating that the participants did not agree these two factors influenced their decision for not doping. Also, there was significant difference in the scores as revealed by Wilcoxon signed test, between Personal Ethical Standards and the remaining factors except Illegality of Substances (z=-1.705, p=0.088). Gender association was noted for anti-doping education and testing, with higher scores in males (p=0.031). Also Type of Main Sport had association with most factors except Long Term Health Implications while Level of Sport did not show any association except for Influence of Significant Others.

**Conclusion:**

Study concludes that Illegality of Substances and Personal ethical standards are the most significant factor for athletes’ decision for not doping.

## INTRODUCTION

The international sports community has witnessed increased hue and cry over doping which is the use of performance enhancing drugs (PED) in sports^1^ that provide an illegitimate advantage to players/athletes. It is an issue of international prominence,^2^ with sports under the umbrella of World Anti-Doping Agency (WADA) being covered under anti-doping laws. Though doping is dangerous with serious health consequences it is usually adopted to obtain unfair advantage in sports.^3^ Not only drugs are used for doping, even dietary energy supplements have been implicated with health hazards like hepatotoxicity.^4^ In spite of the dangerous health hazards associated with supplements, their use is widespread ranging between 40-100% in athletes.^5^ Unfortunately, the menace of doping is sustained by new substances and techniques, in-spite of efforts to curb the menace, even in developing countries^6^ like Pakistan, India, and Bangladesh. The race of winning medals and gaining fame is endless. The lust of prize money may also be the cause behind many other reasons to dope along with the bitter reality that majority of athletes belong to mid and low socio economic strata in developing countries like Pakistan. Deficiencies in screening process, result in involvement of athletes/ sportsmen in doping especially at national championship levels. This is a matter of grave concern not only for trainers and coaches but also a main concern due to long term health hazards, risks and its consequences. The latest changing rules of WADA (world anti-doping agency) and threat of imposing sanctions on athletes is the only deterrent to produce neat clean athletes beside pure anti-doping education.

The menace of doping is increasing day by day in Pakistani sports players especially in power sports. Athletes are unaware of harmful effects of doping on human body which may lead to death. There is a need to analyze the factors which play significant role for athletes towards doping in Pakistan, hence this study was conducted to evaluate the attitude of athletes towards performance enhancement through doping in Pakistan and to determine the major leading reason of their decision for the use of doping in the country.

## METHODS

This cross-sectional descriptive study recruited a sample of n=377 national and international athletes/players using non-probability convenience sampling. Sample size was calculated using Raosoft software with confidence level of 95% and margin of error of 5. Sample included athletes/players of both genders aged 17-35 years recruited from different teams and individual sports, who were camping in Pakistan under the umbrella of Pakistan Sports Board, for preparation of forthcoming 13th South Asian Games scheduled to be held in Dec 2019 in Kathmandu (Nepal). Study was conducted over a period of six months from November 2018 to May 2019 at Pakistan Sports Board, Jinnah Complex Islamabad, Pakistan. Athletes/ players with any disease, trauma or working as coaches or officials were excluded from the study.

Basic demographic sheet and Athletes Attitude to Doping Questionnaire,^7^ were used for data collection with six factors including Personal ethical standards, Illegality of substances, psychosocial influences, Influences of significant others, Anti-doping education and testing and long term health implications. Following ethical approval of research from Ethical Research Committee vide Reg. No. 1709-M.Phil. HPESS-010 dated 29^th^ Nov. 2018, and after obtaining consent, Basic demographic sheet and Athlete Attitude to Doping Questionnaire was applied to the study population by the researcher. A minimum of 30 minutes were given to all respondents to reply the questions in the questionnaire. Responses were recorded on 6 point Linkert scale. 1 = Strongly Disagree, 2= Disagree, 3= Somewhat Disagree, 4= Somewhat Agree, 5=Agree, 6=Strongly Agree.

Data collected was organized in Excel worksheet and SPSS 21.0 used for data analysis. Descriptive and non-parametric tests were used for data analysis. Variables studied included factors of anti-doping questionnaire in addition to gender, type and level of sport, dope education and testing. T-Test and Anova were used to see association, while mean and median were calculated for factors and non-parametric tests like Friedman test & Wilcoxon Signed test used to see associations of factors.

## RESULTS

Of the sample population of n=377 athletes and sportsmen comprised 272 (72.15%) male and 105 (27.85%) female ratio of 2.59: 1 and a mean age of 24.59 ± 2.99 years. The population was involved in 10 varieties of sports with majority involved in martial arts and most were national players (Table-I).

**Table-I:** Demographic Characteristics* Anti-doping Factors. Descriptive statistics and Association. (n=377).

Variable	Characteristic (N)	Personal Ethical Standards	Illegality of Substances	Psychosocial Influences	Influence of significant others	Anti-doping Education and Testing	Long-term Health Implications

		Mean SD	Mean SD	Mean SD	Mean SD	Mean SD	Mean SD
Gender	Male (n=272)	5.08±.66	5.14±0.70	2.99±0.57	4.65±1.16	4.86±0.57	2.09 ±0.88
	Female (n=105)	4.93±1.02	5.13±0.65	3.02±0.68	4.4±1.26	4.7 ± .74	2.28 ± 1.13
	T-test	T=1.697, P=0.091	T=0.034, P=0.973	T=-0.382, P=0.703	T=1.804, P=0.072	T=2.166, P=0.031	T=-1.67, P=0.096
Main Sports	Track and Field Throwers (n=26)	5.35±0.56	5 ±0.69	2.77±0.59	4.96±0.73	4.85 ±0.61	2.27 ± 1.08
	Track and Field Jumpers (n=30)	5.5±0.57	5.33±0.71	2.73±0.78	3.17±1.68	4.57 ±0.93	2.23±1.41
	Track and Field Sprinters (n=44)	5.14±.67	5.07±.62	3.32±0.64	3.52±1.45	4.93 ±0.50	2.39 ± 1.15
	Boxing (n=49)	4.35±1.14	5.16±0.72	2.94±0.47	5.06±0.72	4.92 ±0.49	2.02 ± 0.72
	Wight Lifting (n=51)	5.2±0.56	5.29±.61	3.2±0.60	4.86±0.75	4.92 ± 0.34	2.06 ± 0.73
	Volley ball (n=30)	4.93 ±0.74	5.17±.69	3 ± 0.37	4.03±1.15	4.53 ± 0.73	2.1 ± 0.71
	Martial Arts (n=85)	4.94 ± 0.68	4.95±.78	2.91±0.57	4.89±0.79	4.68 ±0.68	2.16 ± 1.04
	Basket Ball (n=30)	5.13 ±0.51	5.17±.059	2.93±0.58	5 *±*0.78	4.97 ±0.56	1.83 ± 0.79
	Wrestling (n=15)	5.53 ±0.52	5.07±0.46	3.13±0.52	5 ±0.84	5.07 ±0.59	2.13 ± 0.91
	Hockey (n=17)	5.18±0.64	5.47±0.51	3.06±0.66	5.35±0.61	5 ±0.71	2.29 ± 0.77
	Anova Stats.	F=8.542, P=0.000	F=1.929, P=0.047	F=3.705, P=0.000	F=18.085 P=0.000	F=2.844, P=0.003	F=0.025, P=0.503
Level of Sports	National (n=300)	5.06±0.56	5.12±0.69	3 ±0.59	4.6 ±1.14	4.81±0.64	2.14 ±0.94
	International (n=74)	4.99±0.87	5.23±0.65	3 ± 0.62	4.62±1.27	4.85 ± 0.54	2.14 ± 1.04
	Regional (n=3)	5 ± 0	4.67±0.58	3.33±0.58	2.33 ± 2.31	4.67 ± 1.53	3 ± 1
	Anova Stats.	F=0.245, p=0.783	F=1.506, P=0.223	F=0.464, P=0.629	F=5.536, P=0.004	F=0.214, P=0.807	F=1.2, P=0.302
Drug Test in 2 Years	Yes (n=63)	5 ±.89	5.22±0.66	3.02±0.64	4.61±1.26	4.81 ± 0.50	2.11 ± 1.05
	No (n=314)	5.05 ±0.75	5.12±0.69	3 ±0.59	4.58±1.18	4.82 ± 0.65	2.15 ± 0.95
	Anova Stats.	F=0.225, P=0.636	F=1.208, P=.272	F=0.053, P=0.819	F=0.048, P=0.826	F=0.011, P=0.918	F=0.084, P=0.772
Dope Test Result	Negative (n=62)	5 ±0.90	5.23±0.66	3.02±0.64	4.62±1.27	4.81 ± 0.51	2.11 ± 106
	No (n=315)	5.05±0.75	5.12±0.69	3 ± 0.59	4.57±1.18	4.82 ± 0.65	2.15 ± 0.9
	Anova Stats.	F=0.221, P=0.639	F=1.285, P=0.258	F=0.053, P=0.818	F=0.084, P=0.772	F=0.021, P=0.885	F=0.074, P=0.786
	Yes (n=330)	5.03±0.81	5.13±0.70	3.01±0.60	4.61±1.19	4.83 ± 0.62	2.16 ± 0.98
	No (n=47)	5.11±0.52	5.17±0.56	2.96±0.59	4.38±1.17	4.72 ± 0.68	2.02 ± 0.82
	Anova Stats.	F=0.363, P=0.547	F=0.138, F=0.710	F=0.268, P=0.605	F=1.51, P=0.220	F=1.192, P=0.276	F=0.862, P=0.354

The results of the six factors of the anti-doping questionnaire (Table-II) revealed significant difference in the Mean and Median scores for these factors. A very low Mean and Median score for “Long Term Health Implications” (Mean= 2.14, Md=2) and a low score for “Psychosocial Influences” (Mean=3, Md=3) compared to a high score for the remaining factors, indicating that the participants did not agree these two factors influenced their decision for not doping. Friedman’s Test Rank also showed a low Mean Rank for Psychosocial influences and Long-Term Health Implications compared to other factors (p=<0.001). Wilcoxon Signed Rank Test was also applied and results (Table-II) revealed statistically significant difference in the scores between Personal Ethical Standards and the remaining factors except Illegality of Substances (z=-1.705, p=0.088). Also, there was statistically significant difference between scores of the remaining factors with p value of < 0.001.

**Table-II:** Descriptive and Non Parametric Test Results* Six Factors of Anti-Doping Questionnaire. Cross Tabulation (n=377).

Stat. Tests		Antidoping Factors					

		Personal Ethical Standards	Illegality of Substances	Psycho-social Influences	Influence of significant others	Anti-doping Education and Testing	Long-term Health Implications
Mean		5.04	5.14	3	4.58	4.82	2.14
Median		5	5	3	5	5	2
Mode		5	5	3	5	5	2
Range		5	3	4	5	4	5
Friedman Test	Mean Rank	4.6	4.74	1.99	4.04	4.24	1.39
Wilcoxon Signed Test (Z value, P-Value)	Personal Ethical Standards		-1.705b 0.088	-16.548c 0.000	-5.631c 0.000	-4.611b 0.000	-16.701b 0.000
	Illegality of Substance			-16.820b 0.000	-7.028b 0.000	-6.307b 0.000	-16.725b 0.000
	Psycho-social Influences				-14.236b 0.000	-16.753b 0.000	-11.621c 0.000
	Influence of Sig. Others					-2.837b 0.005	-15.715c 0.000
	Anti-doping Education & Testing						-16.618c 0.000

Statistically significant gender association was noted for anti-doping education and testing with higher scores in males (p=0.031). Also, significant association was noted for Type of Main Sport with most factors except Long Term Health Implications. As regards Level of Sport no significant association was noted for most factors except for Influence of Significant Others (P=0.004) with highest score for International (4.62 ± 1.27) followed by National (4.6 ± 1.14) was noted. No significant association was noted for Drug test in two years and Dope Test result and Dope Education with any of the six factors.

## DISCUSSION

The current study revealed a high mean and median score on Anti-doping questionnaire with high scores for Illegality of substance (5.14, 5) and Personal ethical standard (5.04, 5), lower scores for Anti-doping education and testing (4.82,5) and Influence of significant others especially coaches (4.58, 5) and low scores for Psycho-social Influences (3,3) and Long Term Health Implications (2.14, 2).

A low score for “Psychosocial Influences” and “Long Term Health Implications” compared to a higher score for the remaining factors, indicating that the participants did not agree that these two factors influenced their decision for not doping. Hence the most significant factor affecting athlete’s decision making for not doping in Pakistan are Illegality of substances and Personal Ethical Standards.

Similarly, a previous study by MacNamara and Collins, supported that the most significant factor as Personal Ethical Standards followed by Illegality of substances which influence decision making for not doping. This was followed by lesser significant factors including Psycho-social factors, Anti-doping Education, Anti-doping Testing, Long-term Health Implications and family and friends.^7^ It was also interesting to know that in our study psychological and long term health implications were not important factors. This difference may be due to cultural, ethnic and low literacy levels along with scarcity of doping testing for athletes in Pakistan. Elbe & Brand in their study using ethical decision making training found positive results for anti-doping.^8^ Personal factors including moral stance also emerged as a substantial factor that was identified affecting decision not to dope in another study,^9^ while Petroczi, reported that orientation of athletes to competitiveness and to win plays a statistically significant role.^10^ According to Badea DN et al., role of social support factors also needs to be considered since they influence doping decision making.^11^ Coaches have important role and may be involved in illegal actions of athletes by introducing/ offering illegal substances.^12^

In the current study, significant gender association was noted for anti-doping education and testing with higher scores in males (p=0.031). Similarly, different Studies reported higher doping susceptibility in males.^13^,^14^ In contrast in one study personal ethical standards were more important to females (35%) then males (29%), while as regards Long term health implications, males were more concerned (17%) then females (11%).^7^

The present study revealed significant association for type of Main Sport with most factors except Long Term Health Implications. Alaranta A et al. reported highest risk of doping in speed and power sports, and lower in sports demanding motor skills.^14^ Another study reported higher doping susceptibility in swimmers who took dietary supplements.^13^ According to Yildiz Q et al., in opinion of body builders success was difficult without PED’s resulting in use of PED’s in most.^15^ A Korean study reveals that athletes involved in motor skill sports were more inclined toward doping than those in team category.^16^ 17. Morente-Sanchez J et al. in a Spanish study involving cyclists a number of Olympic disciplines in general were against doping, while, Bicycle Moto Cross (BMX) and Track riders seemed more permissive towards the using PEDs than Mountain bike (MTB) and Road.^17^

As regards Level of Sport, current study did not reveal any significant association for most factors except for Influence of Significant Others with highest score for International sports level. In one study higher performance enhancement attitude scale (PEAS) score was noted for High school than middle school athletes; also team sport had lower score then athletes in endurance or motor skill sports.^18^

In current study no significant association was noted for Drug test in two years and Dope Test result and Dope Education with any of the six factors. Also doping masking is advancing ahead of doping detection tests,^12^ hence doping control by tests is not enough requiring an attitudinal change,^14^ with in-depth education regarding anti-doping.^16^,^17^

### Limitation of the study

The athletes had very limited exposure of international competitions except few who fetched medals for Pakistan in Asian and South Asian championships.

## CONCLUSIONS

Illegality of Substances and Personal ethical standards are the most significant factor for athletes regarding decision for not doping.

### Authors` Contribution:

**GSA** designed, did the data collection, statistical analysis and is responsible for integrity of research.

**NM** reviewed and did the final approval of manuscript.

**GS** did the manuscript writing and literature review.
